# Real-time carbon allocation into biogenic volatile organic compounds (BVOCs) and respiratory carbon dioxide (CO_2_) traced by PTR-TOF-MS, ^13^CO_2_ laser spectroscopy and ^13^C-pyruvate labelling

**DOI:** 10.1371/journal.pone.0204398

**Published:** 2018-09-25

**Authors:** Lukas Fasbender, Ana Maria Yáñez-Serrano, Jürgen Kreuzwieser, David Dubbert, Christiane Werner

**Affiliations:** Ecosystem Physiology, Institute of Forest Sciences, Faculty of Environment and Natural Resources, Albert-Ludwigs-University Freiburg, Freiburg, Germany; University of Copenhagen, DENMARK

## Abstract

Our understanding of biogenic volatile organic compound (BVOC) emissions improved substantially during the last years. Nevertheless, there are still large uncertainties of processes controlling plant carbon investment into BVOCs, of some biosynthetic pathways and their linkage to CO_2_ decarboxylation at central metabolic branching points. To shed more light on carbon partitioning during BVOC biosynthesis, we used an innovative approach combining δ^13^CO_2_ laser spectroscopy, high-sensitivity proton-transfer-reaction time-of-flight mass spectrometry and a multiple branch enclosure system in combination with position-specific ^13^C-metabolite labelling. Feeding experiments with position-specific ^13^C-labelled pyruvate, a central metabolite of BVOC synthesis, enabled online detection of carbon partitioning into ^13^C-BVOCs and respiratory ^13^CO_2_. Measurements of trace gas emissions of the Mediterranean shrub *Halimium halimifolium* revealed a broad range of emitted BVOCs. In general, [2-13C]-PYR was rapidly incorporated into emitted acetic acid, methyl acetate, toluene, cresol, trimethylbenzene, ethylphenol, monoterpenes and sesquiterpenes, indicating *de novo* BVOC biosynthesis of these compounds. In contrast, [1-13C]-pyruvate labelling substantially increased ^13^CO_2_ emissions in the light indicating C1-decarboxylation. Similar labelling patterns of methyl acetate and acetic acid suggested tightly connected biosynthetic pathways and, furthermore, there were hints of possible biosynthesis of benzenoids via the MEP-pathway. Overall, substantial CO_2_ emission from metabolic branching points during *de novo* BVOC biosynthesis indicated that decarboxylation of [1-13C]-pyruvate, as a non-mitochondrial source of CO_2_, seems to contribute considerably to daytime CO_2_ release from leaves. Our approach, combining synchronised BVOC and CO_2_ measurements in combination with position-specific labelling opens the door for real-time analysis tracing metabolic pathways and carbon turnover under different environmental conditions, which may enhance our understanding of regulatory mechanisms in plant carbon metabolism and BVOC biosynthesis.

## Introduction

Our understanding on emissions of biogenic volatile organic compounds (BVOCs), including thousands of different compounds from different chemical classes, has improved substantially during the last decades, partially due to new developments in measuring techniques. Therefore, the biosynthetic pathways of a great number of volatile compounds such as isoprenoids, wound induced compounds or several oxygenated compounds (e.g. acetaldehyde, ethanol, methanol) are well—but still not fully—understood. In contrast, very little is known on the underlying biochemical production processes of many other volatiles (e.g. benzenoids, methyl acetate), the regulation of plant carbon investment into such BVOCs, as well as environmental factors controlling biosynthesis and emission of these compounds. Moreover, unknown processes and key determinants controlling BVOC release from terrestrial vegetation account for large uncertainties in our understanding of global carbon cycling [1,2]. Although ^13^C-labelling experiments already improved our knowledge on carbon allocation into BVOCs [3–5], there are still open questions regarding carbon partitioning into plant primary and secondary (e.g. BVOC emissions) metabolism with special regard to the biochemical link between both.

Noteworthy, the complex biosynthetic pathways of many volatiles produced in plants are tightly linked to CO_2_ exchange between the plant and the atmosphere. For example, biosynthesis of monoterpenes and isoprene, the latter being the most important volatile emitted by vegetation, takes place in the chloroplasts and uses Calvin cycle intermediates (pyruvate and glyceraldehyde-3-phosphate) as direct precursors. Hence, production of these compounds is tightly linked to photosynthetic CO_2_ fixation [5,6]. On the other hand, use of the key metabolite pyruvate as a precursor often involves a decarboxylation step during biosynthesis, in which the carboxyl group of pyruvate is released as CO_2_ [7–9]. Thus, many pathways producing BVOCs constitute a source of CO_2_ even in the light, besides the main CO_2_ releasing process in plants, mitochondrial dark respiration. Hence, there are at least four main processes determining the CO_2_ exchange in plants, *i*.*e*. CO_2_ fixation during photosynthesis, photorespiration, CO_2_ generating processes in metabolic pathways and mitochondrial respiration mainly taking place in the tricarboxylic acid (TCA)-cycle. From an analytical point of view, mitochondrial respiration can easily be determined in the absence of light, however, it is at least partially inhibited in the light [7,10–14]. In contrast, analysing CO_2_ evolving processes during the day is tricky due to co-occurring photosynthesis. Moreover, in the light multiple sources of CO_2_ production exist, as during production of BVOCs whose link to CO_2_ production has not been well established so far [15]. Tcherkez et al. (2017) point out that a comprehensive analysis of metabolic fluxes, including partitioning at branching points, is still lacking and that the specific origin of carbon atoms fuelling day-respiration is still uncertain.

To unravel metabolic networks, the use of central metabolites, which are position-specific labelled with stable isotopes, reflects a very effective tool in order to follow the metabolic fate of the tracer into different anabolic and catabolic pathways [15,16]. As mentioned above, pyruvate is a central metabolite tightly linking plant primary and secondary metabolism. In the present study, we therefore fed plants with position-specific labelled pyruvate (pyruvate ^13^C-labelled at the C1 or C2-carbon position). This approach enables tracing the fate of single ^13^C-atoms of pyruvate through metabolic branching points into volatile plant metabolites and CO_2_ and allows to investigate their metabolic pathways.

Preliminary experiments indicated the vast potential of combining proton-transfer-reaction mass spectrometry (PTR-MS) with molecular tracer approaches for better understanding carbon partitioning in plants [17–20]. Because incorporation of tracers into individual volatiles happens at short temporal scales and dynamics are diverse for different compounds, quantification of these processes needs precise and synchronized analytical systems. Therefore, we used highly sensitive proton transfer reaction time-of-flight mass spectrometry (PTR-TOF-MS) with stable isotope CO_2_ infrared laser spectroscopy (IRIS) and combined this technological infrastructure with position-specific molecular labelling. We aimed at analyzing the trace gas emissions of a Mediterranean shrub *Halimium halimifolium* under standardized environmental conditions. On the one hand, Mediterranean shrubs from the *Cistaceae* family possess a very active secondary metabolism resulting in the biosynthesis of a plethora of plant secondary metabolites including many BVOC [21,22]. On the other hand, *H*. *halimifolium* exhibits large variations in day respired δ^13^CO_2_ [8,23,24], suggesting a, so far speculative, link to plant secondary metabolism [15,16,25,26].

In particular, we aim to identify and quantify the range of BVOC emitted from *H*. *halimifolium* shoots, link BVOC emission with day released CO_2_ and explore the underlying pathway. To this end, we take advantage of our system, which enables to trace the fate of ^13^C derived from either [1-13C]-pyruvate (PYR) or [2-13C]-pyruvate into BVOCs and CO_2_ in real-time. We anticipated that traceability of ^13^C in different volatile compounds may help elucidating biosynthetic pathways particularly of less studied compounds that are still unclear or not understood at all.

## Material and methods

### Plant material and labelling experiments

Three-year-old, 50–70 cm high *Halimium halimifolium* L. plants, a Mediterranean drought semi-deciduous shrub, were grown in 3 L plastic pots, filled with two parts of potting soil and one part of sand. Plants were fertilized weekly with one-quarter diluted, modified Hoagland´s fertilizer solution [27]. For the experiments, *H*. *halimifolium* plants were transferred from a greenhouse into the walk-in climate chambers, where they were kept at day/night cycles of 12:12 h, 25:25°C, 60:60% relative humidity and 500 μmol m^-2^ s^-1^ PPFD during light period. Acclimation to these conditions lasted for one week; plants were supplied with tap water according to their demand and were fertilized as given above.

For labelling experiments, branches of *H*. *halimifolium* were placed into self-constructed enclosures (see below) the evening before the measurements maintaining the natural orientation of the branches by adjusting the enclosure position. Ninety minutes before ^13^C-labelling, the branches were cut carefully at the petiole without changing the branch position in the enclosure; they were immediately recut under water and the cut end of the petiole was placed in deionized water. Thereafter (after 90 min) deionized water was replaced by a 10 mM pyruvate solution, *i*.*e*. the solution was fed to the shoot via the transpiration stream. Control branch samples were left in deionized water for the entire duration of the experiment. Pyruvate (Cambridge Isotope Laboratories, Andover, MA, USA) was 99% ^13^C-labelled at either the C1 or C2 carbon position dissolved in deionized water. Throughout the entire experiment, continuous online measurements of the isotopic composition of BVOCs and CO_2_ as well as photosynthetic gas exchange fluxes were conducted. Two branches were alternately investigated to increase the number of replicates in a given period of time. A picture of an attached *H*. *halimifolium* branch inside the enclosure system can be found as supplementary information (S1 Fig). All branches were in a good phenological condition and were not flowering.

### Measurement system

**Fig 1:**
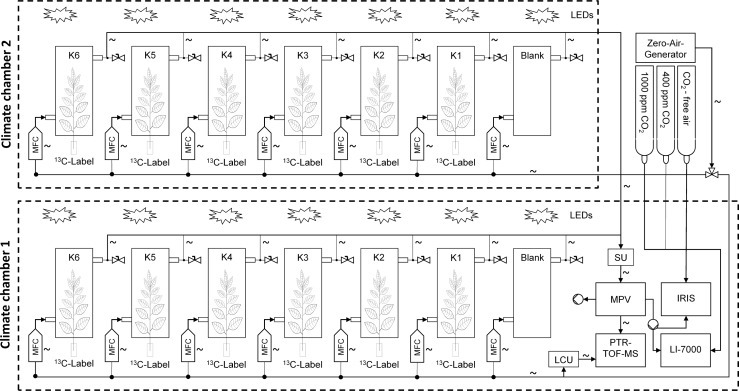
Schematic view of the measurement system. Dotted lines represent the climate chambers. Continuous lines with arrows show the gas tubing and flow direction, while connections are symbolised by a dot. Empty double triangles symbolise valves. Sinuous lines symbolise a heating of the component. “K1-6” stands for enclosures 1–6, “LCU” stands for liquid calibration unit, “MFC” stands for mass flow controller, “MPV” stands for multi-position valve and “SU” stands for switching unit.

We set up a measurement system, consisting of three main units, (i) a zero air generator, providing the experimental system with ultra-pure hydrocarbon free air, (ii) the enclosure system to capture trace gas emissions of intact plants or cut branches, and (iii) the analytical section for simultaneous real-time detection of BVOC, CO_2_ and H_2_O fluxes and their ^13^C isotopes (Fig 1). The analytical section combined, (i) a proton-transfer-reaction time-of-flight mass spectrometer (PTR-TOF-MS, Ionicon Analytic, Innsbruck, Austria), (ii) an isotope ratio infrared spectrometer (IRIS, Thermo Fisher Scientific, Bremen, Germany), and (iii) an infrared gas analyzer (LI-7000 CO_2_/H_2_O Analyzer; LI-COR, Lincoln, NE, USA). To ensure controlled environmental conditions, the plant enclosures and measuring devices were placed within two walk-in climate chambers (ThermoTec, Weilburg, Germany), entirely light, temperature and humidity controlled. Standardized conditions allowed for monitoring of trace gas emissions in response to changes of single environmental parameters such as temperature or light. The climate chambers were illuminated with LED panels with a maximum photon flux density (PFD) of 1600 μmol m^-2^ s^-1^_,_ which generated high and uniform illumination without overheating the enclosures.

We constructed a custom-made zero air generator for continuous supply of hydrocarbon-free, humidified and CO_2_ controlled zero-air at a flow rate of up to 20 l min^-1^. It combined a gas pump, a cold trap, in-line vessels filled with soda lime and activated charcoal, a custom made humidifier and a gas cylinder containing synthetic air mixed with 5 Vol.% CO_2_ (Messer Austria, Gumpoldskirchen, Austria) for CO_2_ enrichment. In addition, the system contained a catalyst module (HPZA-30000, Parker Hannifin Corporation, Lancaster, NY USA) filled with platinum catalyst pellets (Infiltec, Speyer am Rhein, Germany), which efficiently removes nearly all remaining hydrocarbons in the air stream.

The enclosure system consisted seven 600 ml borosilicate glass enclosures (Kummer, Freiburg, Germany), six of them were used as plant enclosures and one was kept empty as a reference. Branches were inserted into the bottomless enclosures and tightly closed with PTFE-foil at the plant stem. The inlet flow was controlled by mass flow controllers (Omega Engineering, Stamford, CT, USA) and kept constant at 600 ml min^-1^ resulting in a calculated residence time in the enclosures of 60 s. The outlet flow was split to channel enclosure air to the different analytical devices. The response-time of air leaving the enclosure and reaching the PTR-TOF-MS and IRIS/IRGA was less than 4 and 10 seconds, respectively. In operation mode, the analytical instruments were supplied with an air flow of ~ 400 ml min^-1^ resulting in a slight overpressure in the enclosures. To assure chemical inertness, all cuvette system parts were made of glass or PFA, and outlet lines were continuously isolated and heated up to 60°C to prevent BVOCs adsorption at the tube walls or water condensation. Inside the PTR-TOF-MS mainly polyetheretherketon (PEEK) and metal is used.

The enclosure air temperature increased by 2°C (from 25±0.5°C to 27±0.5°C) during light period, which is an inhered property of enclosure systems and was taken into account in data analysis. A moderate light reduction of 15–20% was compensated by adjusting the height of the enclosures.

The zero air generator and enclosure system were tested by supplying the empty enclosure system with 1000 ml min^-1^ of purified air. All parameters remained stable during one hour of measurements (CO_2_ 400±1 ppm; H_2_O 15000±500 ppm; e.g. acetone 0.2±0.1 ppb; δ^13^CO_2_−4±0.5 ‰), there were no substantial differences between single enclosures, nor did the enclosure system have any significant influence on the zero air itself. Compared to climate chamber or even bottled hydrocarbon free synthetic air (Messer Industriegase, Bad Soden, Germany), BVOCs were markedly lower after passing the zero air generator, indicating that the instrument efficiently removed BVOCs from ambient air (S2 Fig)

### BVOC detection by PTR-TOF-MS and TD-GC-MS

Online BVOC measurements were conducted with a 4000ultra PTR-TOF-MS (Ionicon Analytic, Innsbruck, Austria). This PTR-TOF-MS version has a built-in internal mass calibration standard (diiodobenzene, m/z: 330.848 and fragments on 203.943), as well as an ion funnel at the end of the drift tube, which focuses the ion beam to the detector allowing BVOCs with higher molecular masses to be analysed more efficiently. We used a multi-position valve controlled by the PTR-TOF-MS software for switching between the enclosures. All sample lines connected to the multi-position valve were continuously flushed to avoid dead end influences.

The PTR-TOF-MS was operated at 2.7 mbar drift pressure, 600 V drift voltage, at an E/N of 120 Td, and drift tube heated to 80ºC. Water impurities were kept on average below 4%, m/z 29.99 was kept on average below 0.2% and oxygen impurities were kept below 6% relative to the primary ions. The mass resolution was 2000 ± 500 m/Δm depending on the compound.

PTR-TOF-MS data post-processing consisted of (i) correction for non-extending and extending dead times as well as the correction for Poisson statistics [28] and iterative residual analysis and cumulative peak fitting [29] using the PTR-TOF Data Analyzer software version 4.48; (ii) normalization of the data to primary ions and water; (iii) background subtraction; (iv) application of calibration factors and (v) synchronization of PTR-TOF-MS data with the IRIS and IRGA data.

We measured non-^13^C-labelled BVOCs and their isotopologues containing one ^13^C-atom (denoted as ^13^C-BVOCs in the following), which were detected at m/z +1. The PTR-TOF-MS technique is a mass-selective technique, thus isomers could be interfering in the measured masses. Therefore, where possible, thermodesorption—gas chromatography mass-spectrometry (TD-GC-MS) was used to verify the results of PTR-TOF-MS. Polar compounds and those with molecular mass <80 amu were not analysed by TD-GC-MS and we assigned these masses to specific compounds according to literature. These compounds were either previously reported to be emitted by vegetation and/or based on mass accuracy of previously reported PTR-TOF-MS measurements. An overview of possible compounds can be found as supporting information (S1 Table). Moreover, specific compounds such as methyl acetate or kaurene emissions by our *H*. *halimifolium* plants were validated in previous studies by TD-GC-MS measurements and calibration standards [22,30].

Calibration of the assigned BVOCs was done either with a multicomponent calibration gas standard (1000 ppb ± 5%, Ionicon Analytic, Innsbruck, Austria), liquid standards or via transmission calculations. For selected compounds (gas and liquid water based calibrations) humidity dependent calibrations were done. Liquid calibrations with water-based solutions were done for acetic acid and methyl acetate (Sigma-Aldrich, Taufkirchen, Germany). For sesquiterpenes, trimethylbenzene, 2-hexenal and cis-3-hexenol (Sigma-Aldrich, Taufkirchen, Germany) hexanal-based solutions were used. All calibrations were performed using the Liquid Calibration Unity (LCU, Ionicon Analytic, Innsbruck, Austria). Information on transmission calculations can be found elsewhere [31].

Furthermore, potential losses of some BVOCs in the system were tested by injecting calibration gas via a PFA-tube into the enclosures under normal operation mode (~ 600 ml min^-1^ inlet flow, ~ 400 ml min^-1^ sample flow). For this purpose, we used compound concentrations of 20 ppb, which was within the magnitude of most of the measured compounds. Comparison of calibration gas measurements (normalized counts per second (ncps)) with and without enclosure system influence revealed that losses for toluene, isoprene, α-pinene, acetone, methanol, acetaldehyde and crotonaldehyde were negligible (S3 Fig). Negligible losses can be assured only for these compounds, but given that also low-volatility compounds such as the volatile diterpene (C_20_) kaurene could be detected (calibrated and cross-validated with GC-MS measurements) [22], makes us confident that strong influences of the measurement system on the considered BVOCs are unlikely. Furthermore, if there were wall losses, the actual emission rates would be even higher than reported.

### CO_2_ detection by IRIS and IRGA

^13^CO_2_ fluxes [nmol min^-1^ m^-2^] were quantified based on the differences in ^13^CO_2_ isotopic composition and their concentration between empty and plant-containing enclosures as analyzed by a Delta Ray Isotope Ratio Infrared Spectrometer (IRIS, Thermo Fisher Scientific, Bremen, Germany). IRIS is an optical analyzer for continuous, online measurements of δ^13^C-CO_2_, δ^18^O-CO_2_ and CO_2_ mixing ratios in sample gas. The analyzer operates at a sample flow rate of 80 ml min^-1^ with a temporal resolution down to one second and a precision of 0.15 ‰. Net fluxes of ^13^CO_2_ (*e*_*13CO2*_) were calculated per projected leaf area (*s*) as:

$$e_{{13CO}_{2}}=\frac{u_{in}}{s}*({{}^{13}c}_{out}-{{}^{13}c}_{in})$$

where *u*_*in*_ is the molar flow of incoming air calculated as:

$$u_{in}=\frac{V}{t}*\frac{p}{R*T}$$

with *V* being the gas volume, *t* being the time, *p* being the gas pressure, *R* being ideal gas constant and *T* being the temperature, and ^*13*^*c*_*out*_ */*^*13*^*c*_*in*_ is the molar fraction of ^13^CO_2_ at the inlet and outlet of the enclosure calculated as:

$${}^{13}c=\left(\frac{(\frac{\delta_{}^{13}{CO}_{2}}{1000}+1)*{\left(\frac{{}^{13}C}{{}^{12}C}\right)}_{VPDB}}{((\frac{\delta_{}^{13}{CO}_{2}}{1000}+1)*{\left(\frac{{}^{13}C}{{}^{12}C}\right)}_{VPDB})+1}\right)*c_{total}$$

where *c*_*total*_ is the mole fraction of total CO_2_ in the enclosure and ^*13*^*C/*^*12*^*C*_*VPDB*_ is the isotope ratio of the international standard *Vienna Pee Dee Belemnite*.

The IRIS is equipped with reference gases of different isotopic compositions (δ^13^C_VPDB_ of -9.7±0.3‰ /-27.8±0.3‰ and δ^18^O_VPDB-CO2_ of -27.2±0.3‰ /-17.5±0.3‰) (Thermo Fisher Scientific, Bremen, Germany) and CO_2_ concentrations (CO_2_ mixing ratio of 408 and 1008 ppm) (Messer, Bad Soden, Germany). The isotope reference of CO_2_ is automatically diluted with synthetic air carrier gas (Messer, Bad Soden, Germany) to the current CO_2_ concentration of the sample gas. Samples were calibrated for isotopic composition throughout the measurements whenever any significant change in the CO_2_ mixing ratio in the sample gas occurred, e.g. during the change between plant and blank cuvette. Additionally, automatic calibration for concentration dependency of the analyzer was conducted every night. To cross-validate IRIS measurements, especially temporal dynamics in isotope signature of leaf dark-respired CO_2_, we used the in-tube incubation technique as described elsewhere [32].

Photosynthetic gas exchange parameters at branch level were quantified using a differential infrared gas analyzer (LI-7000 CO_2_/H_2_O Analyzer; LI-COR, Lincoln, NE, USA), which analyzes the CO_2_ and H_2_O differences between empty and plant containing enclosures. Transpiration rate (*E*) [mmol m^-2^ s^-1^], assimilation rate (*A*) [μmol m^-2^ s^-1^], and stomatal conductance to water vapor (*G*_*H2O*_) [mmol m^-2^ s^-1^] were calculated according to Caemmerer and Farquhar [33]. The IRGA was calibrated at least once per week with synthetic air (0 and 408 ppm; (Messer, Bad Soden, Germany)) for CO_2_ and just before the experiment for H_2_O by a manufacture calibrated GFS3000 (Walz GmbH, Effeltrich, Germany).

## Results

### Incorporation of ^13^C label into BVOCs and respiratory CO_2_

The PTR-TOF-MS revealed a broad spectrum of over 60 different masses emitted by *H*. *halimifolium* branches at a considerable amount under controlled conditions (S2 Table); a selection of the most abundant compounds was identified and listed in Table 1 and a characteristic TOF spectrum is shown as supporting information (S4 Fig). Each detected m/z was assigned to specific BVOCs, which were classified as terpenoids (isoprene, monoterpenes, sesquiterpenes), benzenoids (1.3-cyclopentadiene, benzene, toluene, o-xylene, cresol, trimethylbenzene, ethylphenol), oxygenated BVOCs (methanol, acetone, acetic acid, methyl acetate) and green leaf volatiles (hexenal, hexanal, hexenol). Some of the compounds could be cross-validated by GC-MS analysis (Table 1) others were putatively identified on the basis of literature references (S1 Table) or in case of ethylphenol tentatively identified based on mass accuracy.

**Table 1:** Selection of BVOCs emitted by *H*. *halimifolium*. 5 min-averaged emission rates [pmol m^-2^ s^-1^] of 15 individual plants in the morning (11 a.m.) under controlled conditions. The m/z, assigned species and their molecular mass are given. Calibration was performed via calibration gas (gaseous), liquid solution (liquid) or via transmission. Some compounds were cross-validated via GC and the match factor given (NIST17 mass spectral library), were nd–not detectable in GC spectra, na–not analyzed. The GC Match for monoterpenes and sesquiterpenes refers to α-pinene and β-caryophyllene. n = 15± standard error (SE). Sensitivities are exemplary shown at a humidity level of 8000 ppm and limits of detection (LODs) are set to 3σ.

m/z	Assigned BVOC	Formula	Molec. mass	Calibration	GC Match	Emission rate	Compound class	Sensitivities	LODs
					[%]	[pmol m^-2^ s^-1^]		[ncps/ppb]	[ppb]
51.04	Methanol	CH_4_O(H_2_O)	50.06	Gaseous	na	2098 ± 324	Oxyg. BVOC	1.02	1.84
59.05	Acetone	C_3_H_6_O	58.08	Gaseous	na	9.9 ± 1.2	Oxyg. BVOC	105.5	0.04
61.03	Acetic acid	C_2_H_4_O_2_	60.05	Liquid	na	87.4 ± 40	Oxyg. BVOC	35.8	0.12
67.05	Cyclopentadiene	C_5_H_6_	66.10	Transmission	na	0.04 ± 0.03	Benzenoid	na	na
69.07	Isoprene	C_5_H_8_	68.12	Gaseous	na	9.0 ± 1.4	Terpenoid	20.9	0.07
75.04	Methyl acetate	C_3_H_6_O_2_	74.08	Liquid	na	629.0 ± 209.6	Oxyg. BVOC	44.4	0.05
79.05	Benzene	C_6_H_6_	78.11	Gaseous	na	22.8 ± 6.8	Benzenoid	85.0	0.02
93.07	Toluene	C_7_H_8_	92.14	Gaseous	99.4	16.5 ± 4.2	Benzenoid	87.8	0.02
99.08	2-Hexenal	C_6_H_10_O	98.14	Liquid	nd	0.1 ± 0.05	GLV	61.7	0.03
101.09	Hexanal/Hexenol	C_6_H_12_O	100.16	Liquid	89.7	149.7 ± 68.1	GLV	0.5	2.09
107.08	o-Xylene	C_8_H_10_	106.17	Gaseous	96.1	1.6 ± 0.2	Benzenoid	86.5	0.01
109.1	Cresol	C_7_H_8_O	108.14	Transmission	nd	0.2 ± 0.05	Benzenoid	na	na
121.1	Trimethylbenzene	C_9_H_12_	120.19	Liquid	91.2	2.8 ± 0.1	Benzenoid	107.6	0.01
123.11	Ethylphenol	C_8_H_10_O	122.16	Transmisson	nd	0.1 ± 0.03	Benzenoid	na	na
137.13	Monoterpenes	C_10_H_16_	136.23	Gaseous	98.2	72.3 ± 2.2	Terpenoid	23.6	0.05
205.19	Sesquiterpenes	C_15_H_24_	204.35	Liquid	98.3	16.5 ± 0.4	Terpenoid	4.5	0.31

By far the highest emission rates were detected for methanol (2.1±0.3 nmol m^-2^ s^-1^), methyl acetate (0.6±0.2 nmol m^-2^ s^-1^), acetic acid (0.1±0.04 nmol m^-2^ s^-1^) and monoterpenes (0.1±0.002 nmol m^-2^ s^-1^) (S5 Fig). In general a high variability in BVOC emission rates between plant individuals and between compounds themselves was detected, the former indicated by high standard errors. Especially for acetic acid and methyl acetate highly variable emission rates were found. For methyl acetate, for example, some individuals were high emitters (1.2±0.24 nmol m^-2^ s^-1^ for 10 plants out of 17), while others emitted significantly lower amounts (0.008±0.003 nmol m^-2^ s^-1^ for remaining seven plants). However, for other compounds such as monoterpenes and sesquiterpenes, similar emission rates among all plants were found. Interestingly, we also observed significant emission of compounds which are barely reported in literature, like cresol, trimethylbenzene, ethylphenol, toluene or benzene with flux rates of 0.2±0.05 pmol m^-2^ s^-1^, 2.8±0.1 pmol m^-2^ s^-1^, 0.1±0.03 pmol m^-2^ s^-1^, 16.5±4.2 pmol m^-2^ s^-1^ and 22.8±6.8 pmol m^-2^ s^-1^, respectively. The mean rates of CO_2_ assimilation, transpiration and stomatal conductance were 7.4±0.5 μmol m^-2^ s^-1^, 1.1±0.03 mmol m^-2^ s^-1^ and 113.3±7 mmol m^-2^ s^-1^ respectively.

To shed more light on the biosynthesis pathways of selected BVOC emitted by *H*. *halimifolium*, position-specific labelling of pyruvate was used. As our measurement system is able to distinguish between ^12^C and ^13^C-BVOC fluxes as well as δ^13^CO_2_ emitted, these experiments were set up to test whether BVOC emissions are linked to CO_2_ released during daytime.

**Fig 2:**
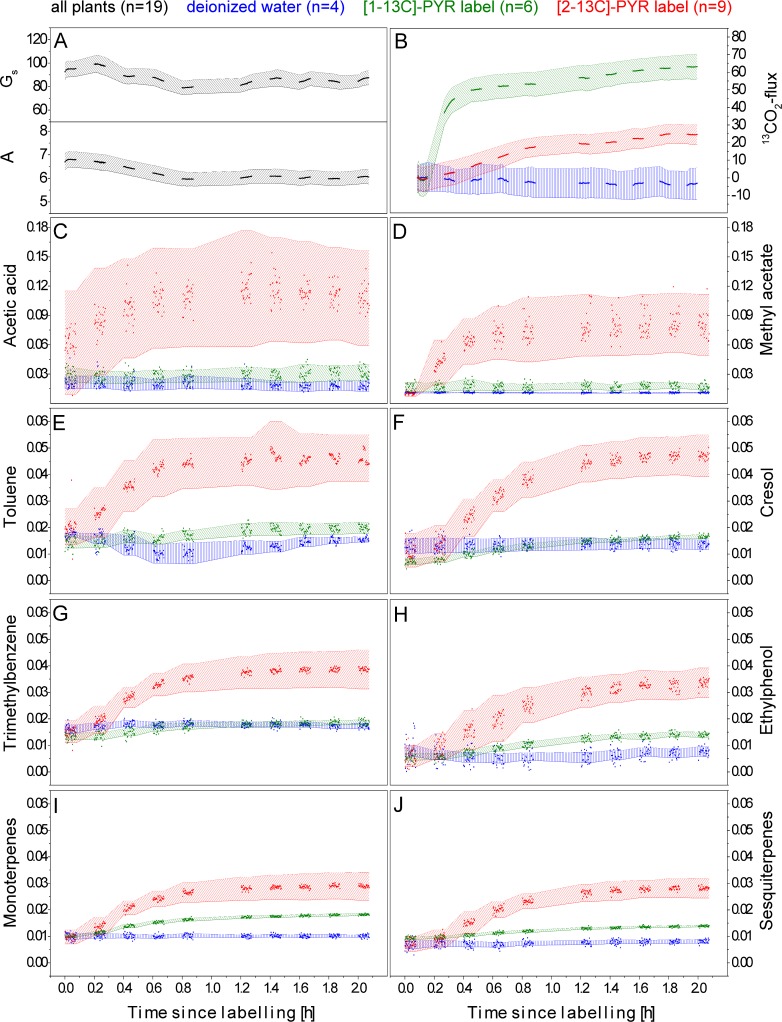
^13^C-label incorporation into BVOC- and respiratory CO_2_ emissions during position-specific ^13^C-pyruvate labelling. A: Stomatal conductance (G_s_) [mmol m^-2^ s^-1^] (top graph) and assimilation rate [μmol m^-2^ s^-1^] (bottom graph). B: ^13^CO_2_-flux [nmol m^-2^ s^-1^]. C-J: ^13^C/C_total_-BVOC ratios of acetic acid (C), methyl acetate (D), toluene (E), cresol (F), trimethylbenzene (G), ethylphenol (H), monoterpenes (I), sesquiterpenes (J). Branches were fed with deionised water (blue symbols), a pyruvate solution ^13^C-labelled at the C1- (green symbols) or the C2 (red symbols) -carbon position. Mean values ± SE (black or coloured shaded area) of n = 19 (black), n = 4 (blue), n = 6 (green) or n = 9 (red) replicates. The labelling started at a time 0 h.

*H*. *halimifolium* branches were fed with either deionised water, [1-13C]- or [2-13C]- position-specific labelled pyruvate solutions (Fig 2, blue, green, and red symbols, respectively), while stomatal conductance (Fig 2A top graph), assimilation rate (Fig 2A bottom graph), ^13^CO_2_-fluxes (Fig 2B) and ^13^C/C_total_-BVOC ratios (Fig 2C–2J) were continuously analysed. Photosynthetic parameter are shown as a mean of all measured plants (n = 19), whereas ^13^CO_2_-fluxes and ^13^C/C_total_-BVOC-ratios are separated by treatment. ^12^C- and ^13^C-BVOC-fluxes can be found as supplementary information (S6 and S7 Figs).

**Fig 3:**
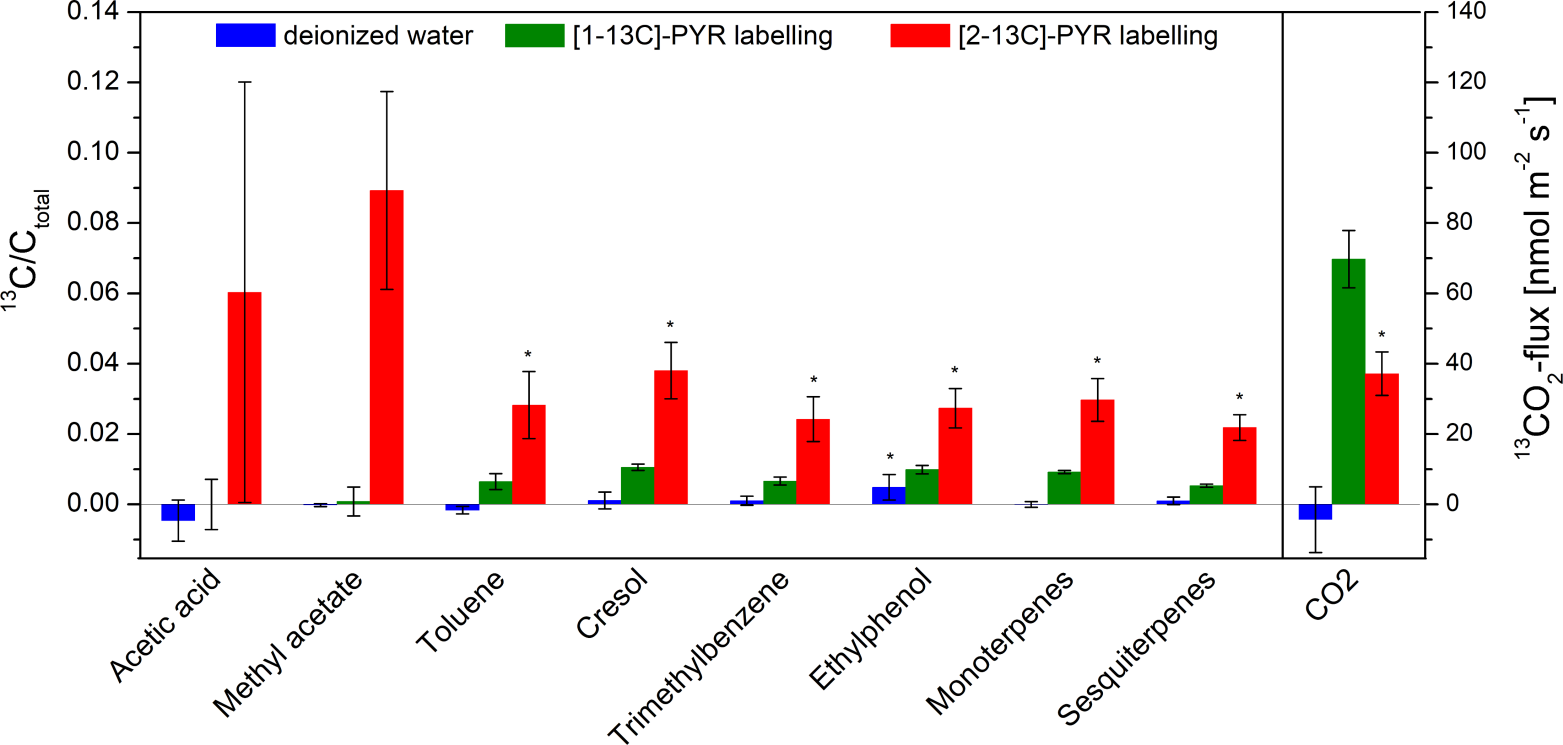
Changes in ^13^C-label incorporation into BVOCs and CO_2_ during 3 h of pyruvate labelling in the light. Branches were fed with deionised water (blue bars), a pyruvate solution ^13^C-labelled at the C1- (green bars) or the C2 (red bars) -carbon position. ^13^C-BVOC fluxes of acetic acid, methyl acetate, toluene, cresol, trimethylbenzene, ethylphenol, montoterpenes and sesquiterpenes are shown in relation to their total carbon fluxes (left axis). Label incorporation into CO_2_ is shown as change in ^13^CO_2_-flux [nmol m^-2^ s^-1^] (right axis). Error bars showing the standard error of n = 4 (blue), n = 6 (green) and n = 9 (orange) replicates. Asterisks indicate significant (P < 0.05) differences (t-test).

In response to cutting, a slight decrease of stomatal conductance and assimilation rates occurred (9 and 11%) during the first 2 hours of labelling. Plants, which did not reach stable values, were discharged from the analysis. The response was similar in control plants (deionized water) and labelled branches, suggesting no appreciable influence of the pyruvate solution. While the ^12^C-BVOC flux of acetic acid (0.14 to 0.15 nmol m^-2^ s^-1^), methyl acetate (0.34 to 0.32 nmol m^-2^ s^-1^) and toluene (0.45 to 0.51 nmol m^-2^ s^-1^) remained constant, the fluxes of cresol (0.002 to 0.006 nmol m^-2^ s^-1^), trimethylbenzene (0.01 to 0.04 nmol m^-2^ s^-1^), ethylphenol (0.001 to 0.003 nmol m^-2^ s^-1^), monoterpenes (0.21 to 0.56 nmol m^-2^ s^-1^) and sesquiterpenes (0.19 to 0.50 nmol m^-2^ s^-1^) increased. In unlabelled control plants the ^13^C/C_total_-BVOC ratio did not change significantly over time (Figs 2C–2J and 3). During [1-13C]-PYR labelling slight but significant increase in the heavier BVOC isotopologue (0.5–1%) was recorded (starting a few minutes after labelling) in almost all considered compounds, indicating a slight progressive incorporation of ^13^C (Figs 2C–2J and 3). However, even after 3 h of labelling, the total incorporation of ^13^C remained minor (<0.1%) in some compounds such as ^13^C-acetic acid and methyl acetate. Since the labelled C1 position was markedly decarboxylated, a significant increase in ^13^CO_2_ emissions was detected in our experiments (Figs 2B and 3). In contrast, [2-13C]-PYR labelling caused rapidly increase of the heavier BVOC isotopologues (Figs 2C–2J and 3), while the ^13^CO_2_ emission during [2-13C]-PYR labelling was threefold lower as compared to [1-13C]-labelling (Fig 2B). Within 3 h of [2-13C]-labelling, significantly increased (2–6%) emission of the heavier BVOC isotopologues were observed for most compounds. As mentioned above, the standard error for acetic acid was high due to large intraspecific variability in the emission rates of individuals for this compound, which, nevertheless, showed an unequivocal incorporation of [2-13C]-PYR label.

**Fig 4:**
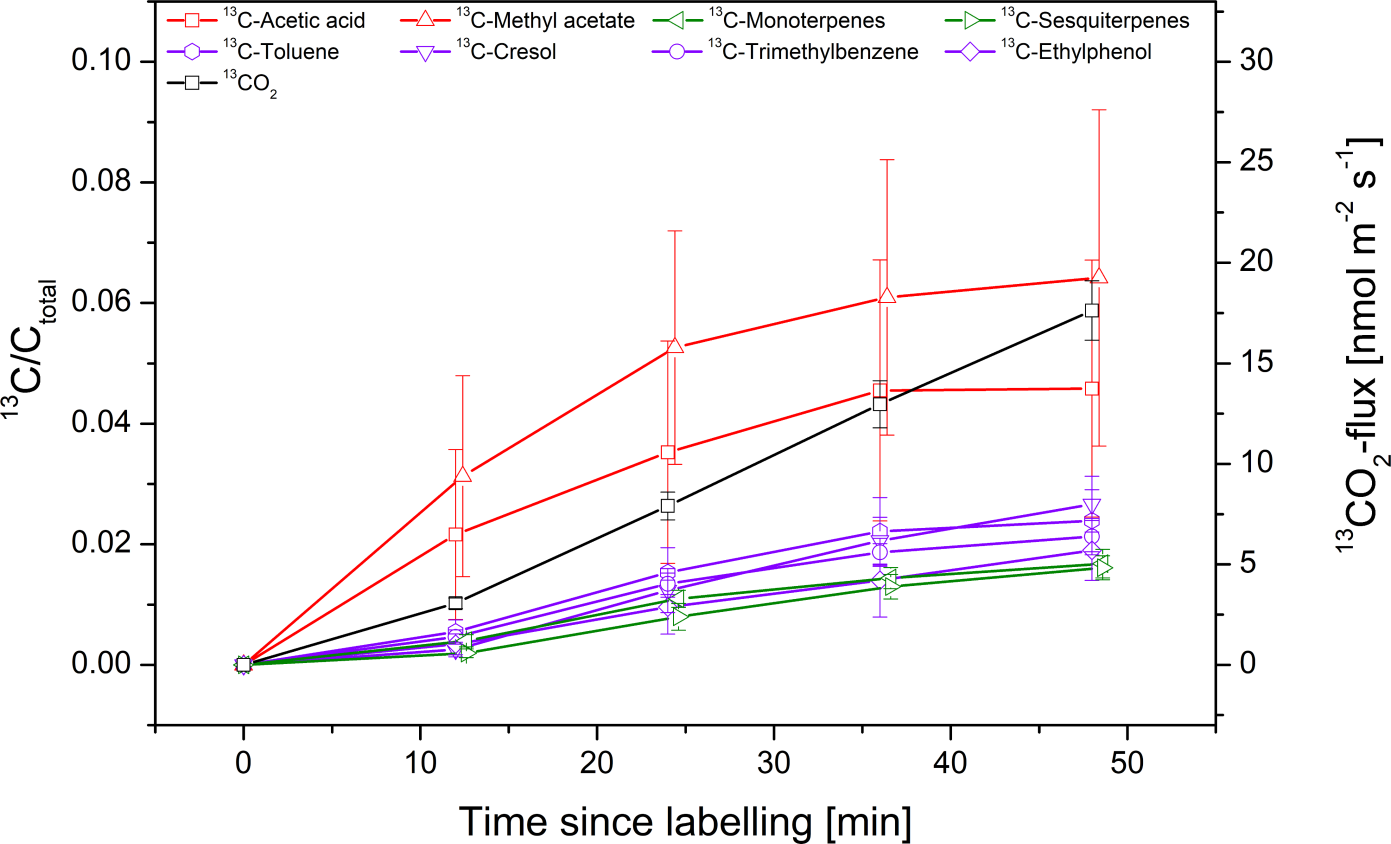
Temporal dynamic of ^13^C-label incorporation into BVOCs and CO_2_ during 50 min of pyruvate labelling in the light. Branches were fed with [2-13C]-position-specific labelled pyruvate. Changes in ^13^C-BVOC-fluxes of acetic acid (red/square), methyl acetate (red/upward triangle), toluene (purple/hexagon), cresol (purple/downward triangle), trimethylbenzene (purple/circle), ethylphenol (purple/diamond), monoterpenes (green/left triangle) and sesquiterpenes (green/right triangle)) are shown in relation to their total carbon fluxes (left axis). Colour code symbolizes the BVOC class. Label incorporation into CO_2_ is shown as change in ^13^CO_2_-flux [nmol m^-2^ s^-1^] (black/square/right axis). Error bars show the standard error of n = 9 individual measurements. All samples are taken at the same time steps; the x-axis shift is just for graphical clearness.

Fig 4 shows the temporal dynamics of the label incorporation into BVOCs and CO_2_ during [2-13C]-PYR feeding. All considered BVOCs showed an increase in the heavier isotopologue already within the first 10 minutes. All benzenoid/terpenoid compounds (Fig 4 purple and green lines) showed an almost linear incorporation, not reaching a steady state within 50 minutes, whereas the oxygenated compounds (Fig 4 red lines) revealed the strongest incorporation in the first 25 min, reaching almost an equilibrium thereafter. We observed clear differences in the velocity (slope) of ^13^C-label incorporation into oxygenated compounds and benzenoids/terpenoids, being fastest into methyl acetate and acidic acid, followed by the others. Interestingly, the terpenoid as well as the benzenoid compounds showed patterns comparable to the [2-13C]-label incorporation into emitted CO_2_ (Fig 4 black line).

## Discussion

In the present study we used position-specific labelling of the key metabolite pyruvate (either [1-13C] or [2-13C]-labelled), which is an efficient tool to trace carbon partitioning at metabolic branching points [15], to elucidate which moiety of the pyruvate molecule is incorporated into volatile compounds or released as ^13^CO_2_ in the light. The latter can be quantified as measurable ^13^CO_2_ emission from decarboxylating enzymatic steps mostly of the [1-13C]-atom of pyruvate. If, however, the entire pyruvate molecule is used as a precursor for volatiles, we expect similar ^13^C-incorporation from both positions. In contrast, if the ^13^C-tracer is only detected after feeding of [2-13C]-PYR, pyruvate must have been decarboxylated and only the C2-C3-moiety of the molecule is used for production of the volatile compound.

In general, labelling via the transpiration stream in cut branches induces mechanical stress to the plant and should therefore not be applied over many hours. Moreover, additional added metabolites to the plant due to labelling might cause changes in metabolic pathways. Therefore, low concentrated labelling solutions were used to reduce the influences and photosynthetic parameters were continuously controlled and remained relatively stable (Fig 2). On the other hand, a great advantage of position-specific metabolite labelling is, that it enables to trace the fate of specific carbon atoms through plant biochemical cycles, a helpful tool to study metabolic branching points. Such position-specific labelling cannot be achieved via fumigation experiments.

**Fig 5:**
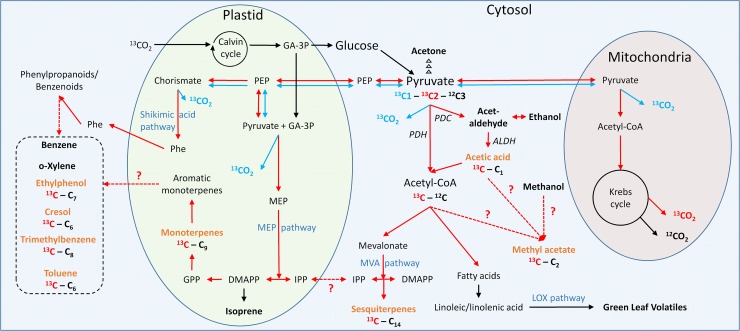
Simplified metabolic scheme of biogenic volatile organic compound (BVOC) biosynthesis in leaves (partly adapted from Dudareva et al. (2006)). Emission rates are given for BVOCs in bold. Bold orange indicates the compounds were we report labelling. Blue arrows symbolize a transfer of the [1-13C]-PYR atom and red arrows symbolize a transfer of the [2-13C]-PYR atom. Dashed arrows symbolize an unclear metabolic pathway. ALDH, aldehyde dehydrogenase; CoA, coenzyme A; DMAPP, dimethylallyl diphosphate; GA-3P, glyceraldehyde-3-phosphate; GPP, geranyl diphosphate; IPP, isopentenyl diphosphate; LOX, lipoxygenase; MEP, 2-C-methyl-D-erythritol 4-phosphate; MVA, mevalonate; PDC, pyruvate decarboxylase; PDH, pyruvate dehydrogenase; PEP, phosphoenolpyruvate; Phe, phenylalanine.

The broad range of volatiles and their isotopoloques emitted from the Mediterranean shrub *Halimium halimifolium* indicated a rapid incorporation of ^13^C to a different extend for each labelled carbon position, which elucidating the production pathways of certain compound groups (Fig 5), as discussed below in detail. Overall, the mass spectra revealed a broad range of organic compounds (> 30) emitted by *H*. *halimifolium* (S2 Table and S4 Fig), a selection of them could be identified and quantified in this study (Table 1). We chose the compounds with highest emission rates, clearest peak for ^12^C- and ^13^C-mass and thorough calibration. The list does not claim completeness but shall give an idea of compounds that are getting labelled via pyruvate during *de novo* biosynthesis. Apart from the well-studied and usually strongly emitted BVOCs like isoprene, monoterpenes, sesquiterpenes, acetaldehyde, formaldehyde and methanol, *H*. *halimifolium* shoots also released less well studied compounds such as methyl acetate and several benzenoids (benzene, toluene, o-xylene, cresol, trimethylbenzene and ethylphenol).

### Biosynthesis of short-chained oxygenated volatiles

We observed a fast incorporation of ^13^C into short-chain molecules such as acetic acid after feeding the plants with [2-13C]-PYR, which was partially released into the atmosphere due to its high volatility. Such connection of pyruvate with formation and emission of oxygenated compounds has been described in several studies [18,19,34]. The ^13^C-labelled pyruvate fed to the plant is transported to the leaves via the transpiration stream where it is taken up by leaf cells [19]. In the cytosol, pyruvate can be converted to acetyl-CoA or acetaldehyde as catalyzed by pyruvate dehydrogenase (PDH) and pyruvate decarboxylase (PDC), respectively [3,18]. Both reactions involve decarboxylation of pyruvate accompanied by a release of CO_2_. Studies causing enhanced acetaldehyde levels in mesophyll cells demonstrated that this toxic compound is further oxidized by aldehyde dehydrogenase (ALDH) to yield acetate [18]. On the other hand, acetate can also be produced during the exchange of C_2_-units between organelles like mitochondria and chloroplasts by acetyl-CoA hydrolysis [35]. Elucidation of the main source of acetate would need further knowledge on the ^13^C-labelling of acetaldehyde, which, unfortunately, was not provided by our data.

While the biosynthesis of acetic acid via acetaldehyde and acetyl-CoA is well described [3,17,36], much less information is available on the formation of the volatile ester methyl acetate in plants [30]. *H*. *halimifolium* strongly emitted this compound at rates of 0.6±0.2 nmol m^-2^ s^-1^, which was an order of magnitude higher than mono- (0.07±0.002 nmol m^-2^ s^-1^) or sesquiterpenes (0.01±0.0004 nmol m^-2^ s^-1^) emission and a little higher than reported rates for this species (0.39±0.22 nmol m^-2^ s^-1^) [30]. Noteworthy, methyl acetate (and also acidic acid) emission rates in our study were quite variable between plant individuals in contrast to other emitted compounds (Fig 2). *H*. *halimifolium* has been characterized as a species with high phenotypic and genotypic plasticity [27,37,38], however, though not being the focus of this study, we were not able to clearly assign high and low emission pattern to individual plants. Since the plants were kept under identical conditions in a controlled climate chamber, we cannot currently disentangle its reasons.

Our labelling patterns suggest that methyl acetate was either derived directly from acetic acid or it was formed from acetyl-CoA, which was produced by conversion of pyruvate and/or activation of acetic acid [30,36,39]. Early work suggested that volatile esters can be synthesised from acetyl-CoA by a transfer of the acetyl moiety to an alcoholic substrate as catalysed by an alcohol acetyltransferase [40]. It has also been proposed that such esters are produced from the lipoxygenase (LOX) pathway, which includes oxidation of cell membrane constituents [41,42]. However, this pathway seems unlikely considering the fast incorporation of the [2-13C]-PYR label in the present study (Fig 4); as we assume that the turnover rates of cell membrane components are much slower. Therefore, the results of our study suggest a link of methyl acetate biosynthesis with the production and emission of acetic acid and confirm earlier assumptions that either acetate or acetyl-CoA are enzymatically methylated [40]. Furthermore, tight linear correlation between ^12^C- and ^13^C-fluxes of acetic acid and methyl acetate (R^2^ = 0.92 and R^2^ = 0.97) support a strong metabolic connection between both compounds (S8 and S9 Figs).

### Metabolic link between the biosynthesis of terpenes and volatile aromatics

Besides these short-chained oxygenated compounds, we observed emission and ^13^C-incorporation into several larger volatile compounds including aromatics and terpenes (Figs 2 and 3). Interestingly, the experiments revealed incorporation of ^13^C into sesquiterpenes, which has been barely reported before. It is generally assumed that sesquiterpenes are mainly synthesised in the cytosol via the mevalonate pathway (MVA) [43], although there are also some reports about a production in the chloroplasts via the methylerythrol-phosphate (MEP) pathway [44,45]. As expected from the intermediates used in these pathways (MVA: acetyl-CoA, MEP: pyruvate), sesquiterpenes showed a strong incorporation of the C2-C3 moiety of the pyruvate applied. The ^13^C from [2-13C]-PYR is used in both pathways to form DMAPP and subsequently farnesyl pyrophosphate (FPP), the universal precursor of all sesquiterpenes [46,47], which ultimately results in biosynthesis of ^13^C-labelled sesquiterpenes [43,48]. In contrast, the weak but distinct incorporation of ^13^C from [1-13C]-PYR may be explained by photosynthetic re-fixation of emitted ^13^CO_2_ [17]. The fixed ^13^CO_2_ contributes to production of glyceraldehyde-3-phosphate, which can be transported to the cytosol, is incorporated into pyruvate derived from glycolysis and subsequently leads to formation of ^13^C-labelled sesquiterpenes. Furthermore, crosstalk between MEP- and MVA-pathway via IPP transport into the cytosol can provide an alternative explanation as well [49,50]. Nevertheless, the quick incorporation of the ^13^C-tracer into sesquiterpenes suggests that these compounds were at least partially produced from *de novo* biosynthesis, which is also supported by our recent study on the same species [22] and is consistent with other work [44,51–53]. However, we detected sesquiterpenes (mainly β-caryophyllene and farnesene) also in storage pools of *H*. *halimifolium* leaves [22], which indicates that emission is driven by both, a temperature dependent release from stored compounds plus *de novo* production of sesquiterpenes.

Similar to sesquiterpenes, we found strong ^13^C-labelling of emitted monoterpenes if [2-13C]-PYR was fed to the plants, and a weaker ^13^C-signal if [1-13C]-PYR was applied. Again, such labelling patterns suggest that the pyruvate taken up by the mesophyll cells was channelled into the plastids and used in the MEP-pathway to form geranyl diphosphate (GPP), the precursor of monoterpenes [43,54]. The MEP pathway starts with the formation of 1-deoxy-d-xylulose 5-phosphate (DXP) by an acyloin condensation of hydroxyl-ethyl-thiamine, derived from the decarboxylated pyruvate, with the C1-aldehyde group of glyceraldehyde 3-phosphate (GA-3P) [49,55]. Thus, due to decarboxylation of pyruvate, the [1-13C]- atom of pyruvate was released as ^13^CO_2_ and could only be used for terpene biosynthesis after photosynthetic re-fixation. Because of the strong ^13^C-labelling of monoterpenes, we further conclude that emission is at least partially driven by *de novo* biosynthesis. In good agreement with this assumption, we recently demonstrated that many volatile terpenes (in the order of abundance ρ-cymen-8-ol > α-pinene > eugenol > limonene > terpinene-4-ol > camphene > p-cymene > r-cymene > myrcene) were present in leaf tissue of *H*. *halimifolium* and many of them seemed to be produced *de novo* in the leaves of this species [22]. Noteworthy, a major portion of the terpenes found in *H*. *halimifolium* leaves were aromatics such as p-cymen-8-ol, eugenol, p-cymene and m-cymene. Radio-labelling experiments and enzymatic evidence suggest that the aromatic monoterpene p-cymene is produced by an aromatization of γ-terpinene [56]. This compound together with the structurally very similar and biochemically related p-cymene-8-ol and terpinen-4-ol was highly abundant in *H*. *halimifolium* leaves [22], suggesting a closely linked production.

Besides terpenes, *H*. *halimifolium* leaves also emitted many aromatic volatiles such as benzene, toluene, trimethylbenzene, o-xylene, cresol and ethylphenol. The emission rates of benzene (22.8±6.8 pmol m^-2^ s^-1^) and toluene (16.5±4.2 pmol m^-2^ s^-1^) were in the same range than sesquiterpene emissions. In contrast, release of trimethylbenzene (2.8±0.1 pmol m^-2^ s^-1^), o-xylene (1.6±0.2 pmol m^-2^ s^-1^), cresol (0.2±0.05 pmol m^-2^ s^-1^) and ethylphenol (0.1±0.03 pmol m^-2^ s^-1^) was considerably lower. The emission rates of toluene observed in the present study were one order of magnitude higher than toluene release of unstressed *Helianthus annuus* leaves (2 pmol m^-2^ s^-1^), but lower than emissions from stressed sunflower plants (up to 100 pmol m^-2^ s^-1^) [57]. Consistent with our data, also other studies reported emissions of benzene, toluene, xylene, trimethylbenzene, cresol and phenol from terrestrial vegetation [57–62]. Only ethylphenol emission from plants was not reported before.

The labelling patterns observed for these compounds (Figs 2 and 3) indicate incorporation of ^13^C from [2-13C]-PYR, whereas, similar to mono- and sesquiterpenes, the [1-13C]-PYR label was not intensively incorporated into volatile benzenoids. The fast incorporation of [2-13C]-PYR label into benzenoids suggest that these compounds are produced *de novo* and emission is not mainly driven from storage pool release. Earlier ^13^CO_2_ fumigation experiments confirm this assumption at least for toluene [57,58].

Even though benzenoids are assumed to be synthesised in the shikimic acid pathway [47,54], the exact biosynthesis pathway of the selected compounds is still unclear [57,58,60]. Importantly, the shikimic acid pathway starts with the addition of pyruvate to erythrose-4-phosphate to eventually form shikimate. In subsequent reactions another PEP is added to shikimate-3-phosphate to generate chorismate. This compound, therefore, should contain two ^13^C-atoms independent if [1-13C]-PYR or [2-13C]-PYR is fed to the plants. Before chorismate is converted to phenylalanine, the assumed precursor for volatile benzenoids, one CO_2_ is decarboxylated either by arogenate dehydrogenase or by prephenate dehydratase (PDT), so that phenylalanine would either be double-labelled with ^13^C if [2-13C]-PYR is fed, or single ^13^C-labelled if [1-13C]-PYR is fed. The aromatic amino acid phenylalanine is converted to cinnamic acid by the action of phenylalanine ammonia lyase (PAL); volatile benzenoids are then produced by further changes and substitutions on the aromatic ring. Importantly, these modifications involve the release of one ^13^C from cinnamate most likely leading to benzenoids, which are labelled by one ^13^C if [2-13C]-PYR was fed and without ^13^C-label if [1-13C]-PYR was fed. Our results support the idea that volatile benzenoids are formed via the shikimate pathway. On the other hand, they show a very close correlation of benzenoids ^13^C-labelling with the labelling of terpenes (Fig 4), a high abundance of aromatic terpenes in leaves of *H*. *halimifolium*, and a very similar chemical structure of these aromatic monoterpenes with the released benzenoids. This highlights the possibility that these compounds might also be derived from the MEP pathway as suggested by Misztal et al. [58]. This hypothesis is supported by the correlation matrix of benzenoids and monoterpenes fluxes (^12^C-monoterpenes to ^12^C-toluene (R^2^ = 0.98), ^13^C-monoterpenes to ^13^C-toluene (R^2^ = 0.99), to ^13^C-cresol (R^2^ = 0.99) or to ^13^C-trimethylbenzene (R^2^ = 0.99)) (S8 and S9 Figs), indicating a strong connection between these biosynthesis pathways. However, further labelling studies of important intermediates, such as phenylalanine or shikimate, is required to elucidate the pathways responsible.

### Day respiratory CO_2_ efflux from metabolic branching points

In general, feeding of [1-13C]-labelled pyruvate resulted in substantially increased ^13^CO_2_-release from the shoots. This increase was threefold stronger as compared to [2-13C]-PYR labelling and detectable immediately after applying the label (Fig 2B), indicating the quantitative relevance of decarboxylation processes during metabolic pathways in plants. As pointed out above, the incorporation of the [1-13C]-atom of pyruvate into the emitted BVOCs was much weaker than the [2-13C]-atom, suggesting that the entire pyruvate molecule is generally not directly used as a precursor of volatile compounds. The remaining weak ^13^C-label may originate from photosynthetic re-fixation of the decarboxylated gaseous ^13^CO_2_. Similar CO_2_ re-fixation and incorporation into volatiles has been reported in ^13^C-labelling experiments with other plants [17,19,63]. However, it must be denoted that incorporation of ^13^C into acetic acid and methyl acetate during [1-13C]-PYR feeding was minor, which is not in line with substantial re-fixation, which would lead to measurable incorporation into these oxygenated compounds. A possible reason for the stronger labelling of e.g. monoterpenes could be the metabolic and spatial proximity to the location of CO_2_ fixation in the Calvin cycle. Acetaldehyde, however, a direct precursor of acetic acid, was found to be strongly labelled during ^13^CO_2_ fumigation of *Prosopis veluntina* [64]. Thus, freshly fixed ^13^CO_2_ is incorporated into oxygenated volatiles, suggesting [1-13C]-PYR labelling of terpenoids and benzenoids might not just be explained by re-fixation.

Moreover, the strong release of ^13^CO_2_ after [1-13C]-PYR feeding indicates that decarboxylation of pyruvate is not restricted to dark period. Thus, this non-mitochondrial source of CO_2_ seems to contribute considerably to the CO_2_ release of leaves during the light period. This observation underlines the importance of CO_2_ production during the day, which is still a highly debated topic [12,65]. It is widely accepted that mitochondrial respiration is partly inhibited in the light compared to the dark due to a reorchestration of major pathways, resulting in a lower CO_2_ efflux [10–14]. But it is also assumed that day respiration can account e.g. for at least 5% of the net assimilation in leaves [13]. Though, at the first glance, it might be of minor importance for a total plant carbon balance, nevertheless under unfavourable environmental conditions with low assimilation rates, day respired CO_2_ may become an important player in the carbon budget [12]. Nevertheless, since the measurement of day respiratory CO_2_ efflux is a nontrivial problem due to strong interaction with photosynthesis and photorespiration, quantifying the amount of day released carbon remains challenging. Hence, precise estimations of day respiratory CO_2_ efflux from plants is still scarce [13] and even less is known on its regulation under changing environmental conditions. Here, we can show that one source of non-mitochondrial CO_2_ release in the light is the decarboxylation of the C1-atom of pyruvate during biosynthesis of a plethora of plant secondary metabolites such as BVOCs, a carbon source which our approach can help to account for.

## Conclusion and perspective

Real-time, synchronised analysis of BVOCs and δ^13^CO_2_ in combination with position-specific labelling of central metabolites provide an enormous potential for biochemical research. Here we demonstrated that position-specific pyruvate labelling combined with on-line BVOC and ^13^CO_2_ measurement helps to disentangle possible biosynthetic pathways of less studied BVOCs such as methyl acetate or toluene. Methyl acetate might have a biochemical origin strongly connected to acetic acid and methanol and there are hints for a synthesis of some benzenoids via the MEP-pathway. Furthermore, during many of these BVOC biosynthesis pathways the C1-carbon atom of the central metabolite pyruvate is decarboxylated, while the remaining C2-C3-moiety is incorporated. Given the fact, that day respiratory CO_2_ efflux is still a big uncertainty in the plant carbon budget, our approach can provide new insights in key determinants controlling the emission of CO_2_ in the light period. Despite of increasing awareness of fast reactions in plant emitted trace gases and a tight linkage between primary and secondary carbon metabolism, there is still a lack of understanding the regulation of carbon allocation into these pathways and driving factors, environmental constrains or differences among plant functional groups. Further investigations opens the door for real-time analysis tracing metabolic pathways and carbon turnover under different environmental conditions, which may enhance understanding of regulatory mechanisms in plant carbon metabolism.

## Supporting information

S1 Table Alternative BVOCs for the measured m/z according to literature [58,64,66–78]. (TIF)

S2 Table Table of detected m/z during labelling experiments. Labelling experiments (L3-L12) for [1-13C]-PYR labelling (C1) or [2-13C]-PYR labelling (C2). Table contains m/z that are clearly distinguishable from background.
(TIF)

S1 Fig Photograph of a typical *H. halimifolium* branch inside the cuvette system. (JPG)

S2 Fig Efficiency of zero-air-generator to purify ambient air. The acetone mixing ratio [ppb] (8 min measurement) in enclosure 1–7 (red to light blue), flushed with 1000 ml min^-1^ of purified air. 25 min measurement of synthetic air (dark blue), supplied from gas vessel (Messer Austria, Gumpoldskirchen, Austria), and 15 min measurement of air in climate chamber (purple). The efficiency is exemplary shown for acetone; most of the other BVOCs revealed similar pattern.
(TIF)

S3 Fig BVOC losses due to cuvette system influences. PTR-TOF-MS measurements (ncps background subtracted) of calibration gas (Ionicon Analytic, Innsbruck, Austria) with or without passing the cuvette system.
(TIF)

S4 Fig Characteristic PTR-TOF-MS spectrum of *H. halimifolium*. Figure shows an averaged (5 min) spectrum of detected BVOCs of one individual plant in the morning (11 a.m.) before a labelling experiment. Spectra shows uncalibrated, background subtracted values in counts per second (cps) plotted by PTR-MS Viewer 3 (Ionicon Analytic, Innsbruck, Austria).
(TIF)

S5 Fig Proportion of identified BVOCs (Table 1) in respect to their sum. (TIF)

S6 Fig ^12^BVOC-fluxes during position-specific ^13^C-pyruvate labelling. Fluxes of acetic acid, methyl acetate, toluene, cresol, trimethylbenzene, ethylphenol, monoterpenes and sesquiterpenes are shown in nmol m^-2^ s ^-1^. Symbols show mean values ± SE (black hatched area) of n = 19 replicates. The labelling started at a time 0 h.
(TIF)

S7 Fig ^13^C-label incorporation into BVOC and respiratory CO_2_ emissions during position-specific ^13^C-pyruvate labelling. A: ^13^CO_2_-flux [nmol m^-2^ s^-1^]. B-I: ^12^C-BVOC fluxes [nmol m^-2^ s^-1^] (black lines; left axis) and ^13^C-BVOC fluxes [nmol m^-2^ s^-1^] (coloured lines; right axis), of acetic acid (B), methyl acetate (C), toluene (D), cresol (E), trimethylbenzene (F), ethylphenol (G), montoterpenes (H) and sesquiterpenes (I). Branches were fed with deionised water (left panel; A1-I1), a pyruvate solution labelled at the C1- (middle panel; A2-I2) or the C2 (right panel; A3-I3) -carbon position. Symbols show mean values ± SE (black or coloured hatched area) for n = 4 (left), n = 6 (middle) or n = 9 (right) replicates. The labelling started at a time 0 h. The proportion between the light ^12^C-BVOC isotopologue (left axis) and the corresponding ^13^C-isotopologue (right axis) is in accordance to the expected proportion of the natural abundance of both isotopologues (Sensu Isotope library, PTR-MS Viewer, Ionicon Analytic, Austria).
(TIF)

S8 Fig Correlation matrix of ^12^C-BVOC fluxes during 3 h of labelling. (TIF)

S9 Fig Correlation matrix of ^13^C-BVOC fluxes during 3 h of labelling. (TIF)
