# Supplementary figures and images for: Real-time carbon allocation into biogenic volatile organic compounds (BVOCs) and respiratory carbon dioxide (CO2) traced by PTR-TOF-MS, 13CO2 laser spectroscopy and 13C-pyruvate labelling
Source: PLoS One. 2018 Sep 25;13(9):e0204398. doi: 10.1371/journal.pone.0204398 (PMC6155514; doi:10.1371/journal.pone.0204398)

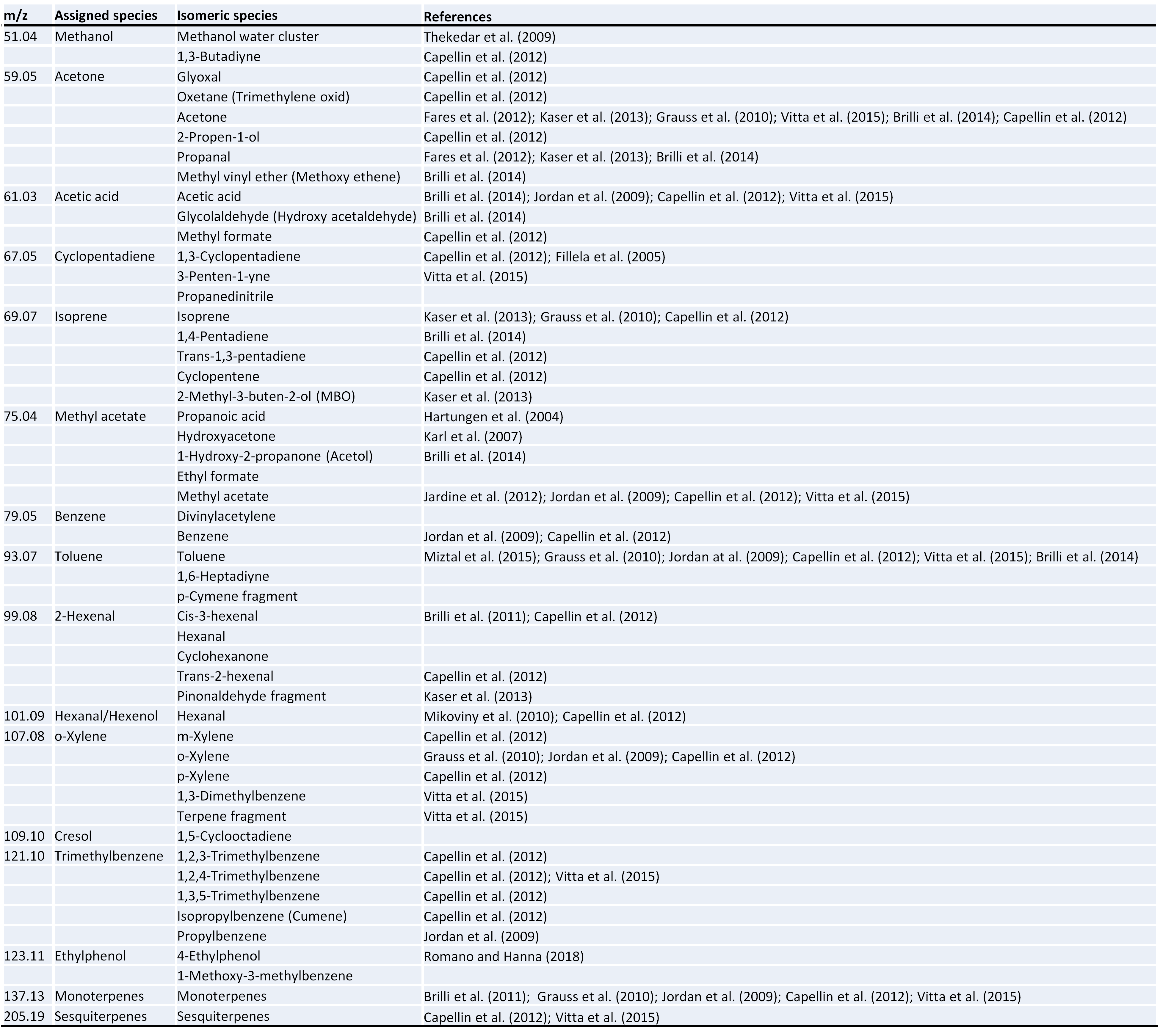

Supplement: S1 Table — (TIF) [file pone.0204398.s001.tif]

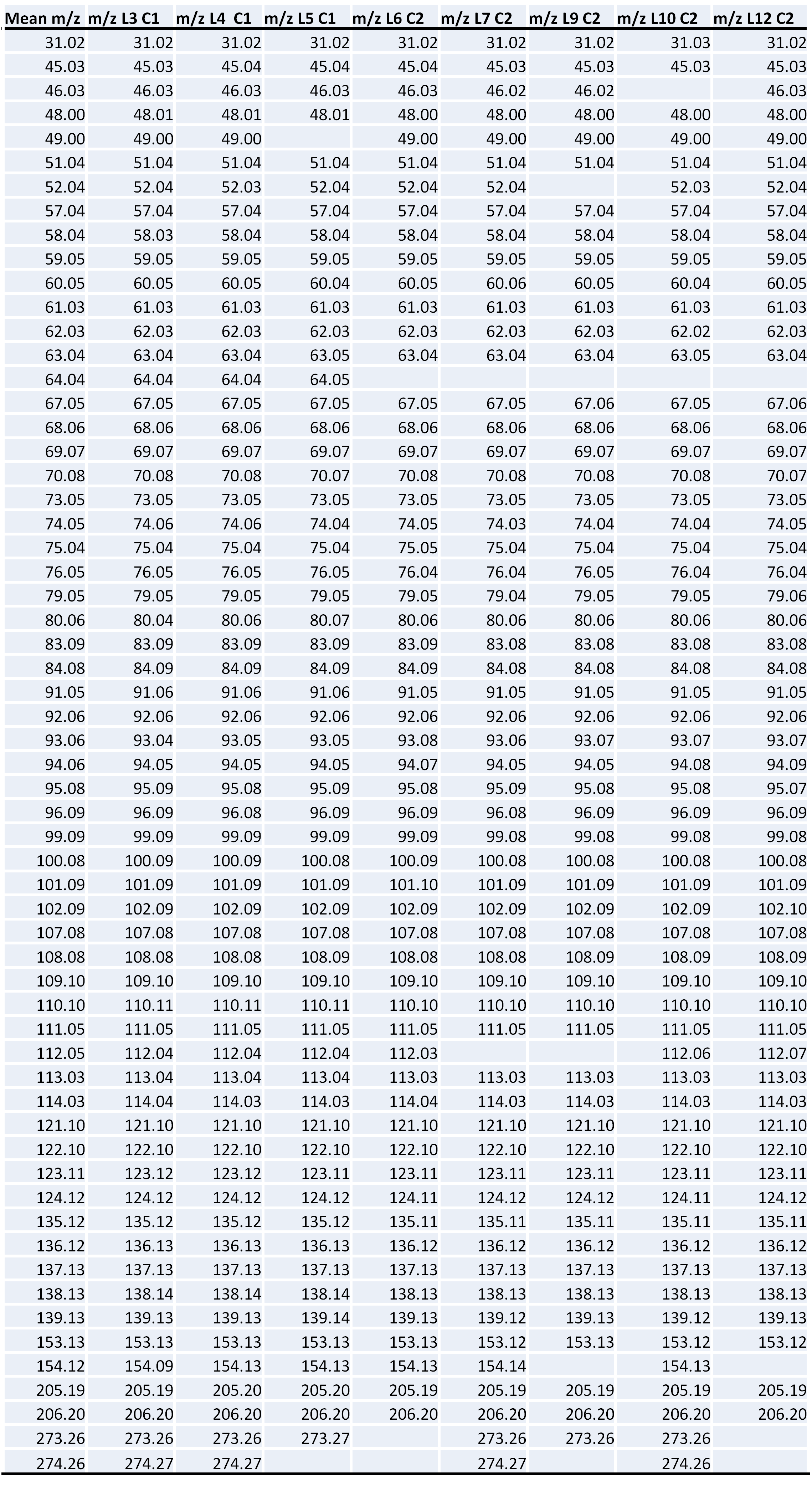

Supplement: S2 Table — Labelling experiments (L3-L12) for [1-13C]-PYR labelling (C1) or [2-13C]-PYR labelling (C2). Table contains m/z that are clearly distinguishable from background. (TIF) [file pone.0204398.s002.tif]

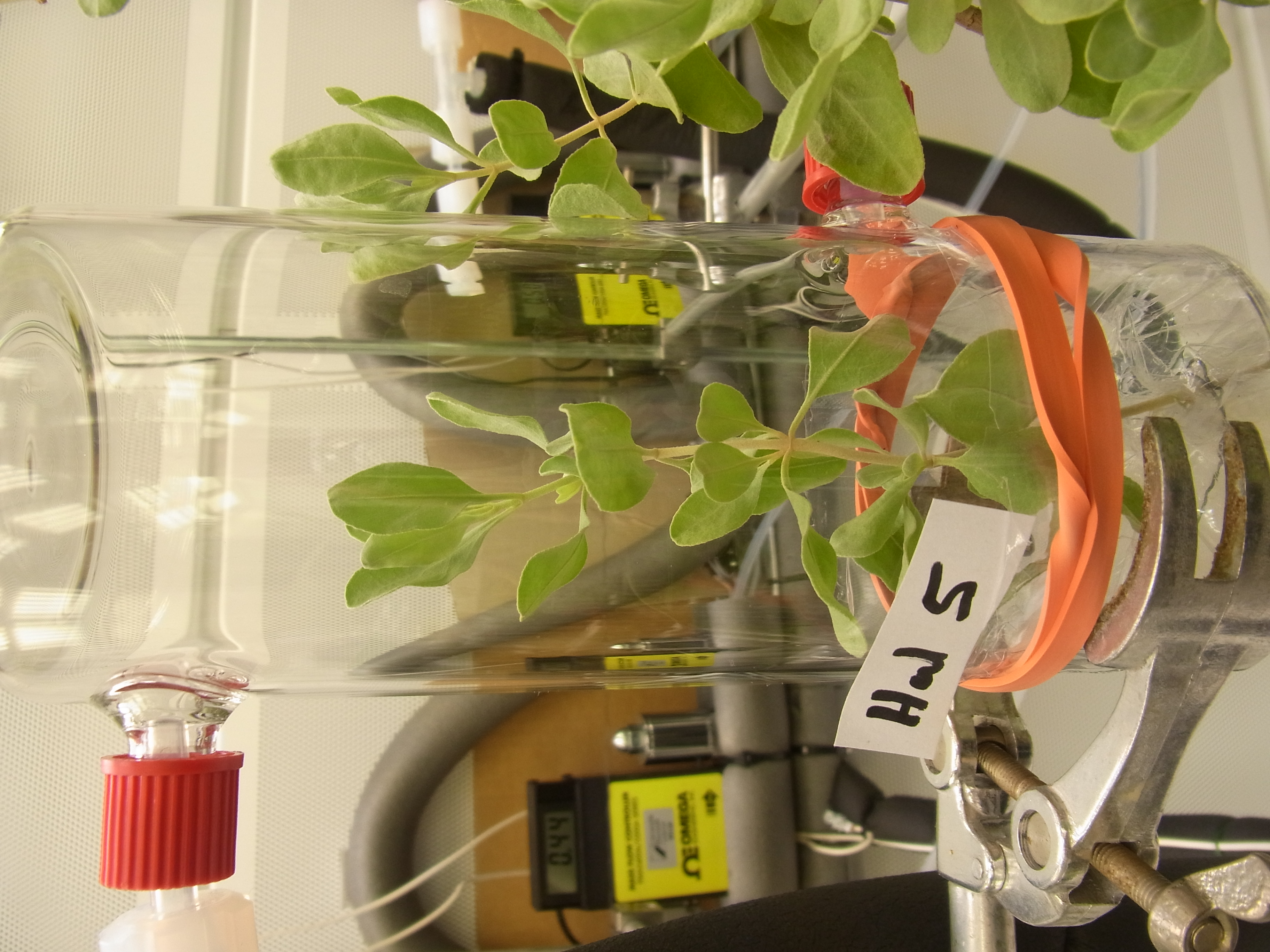

Supplement: S1 Fig — (JPG) [file pone.0204398.s003.jpg]

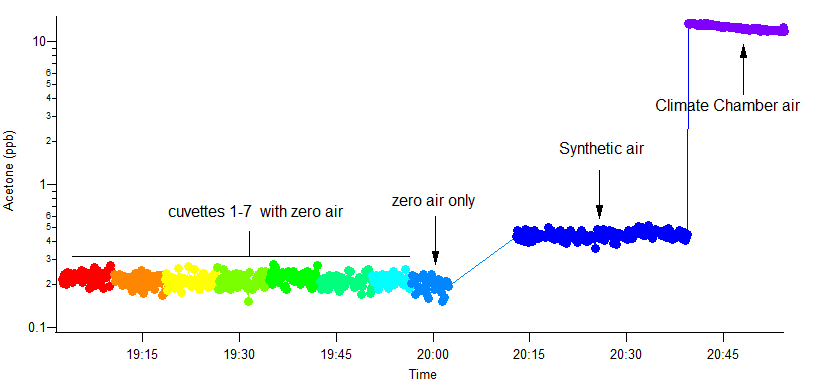

Supplement: S2 Fig — The acetone mixing ratio [ppb] (8 min measurement) in enclosure 1–7 (red to light blue), flushed with 1000 ml min-1 of purified air. 25 min measurement of synthetic air (dark blue), supplied from gas vessel (Messer Austria, Gumpoldskirchen, Austria), and 15 min measurement of air in climate chamber (purple). The efficiency is exemplary shown for acetone; most of the other BVOCs revealed similar pattern. (TIF) [file pone.0204398.s004.tif]

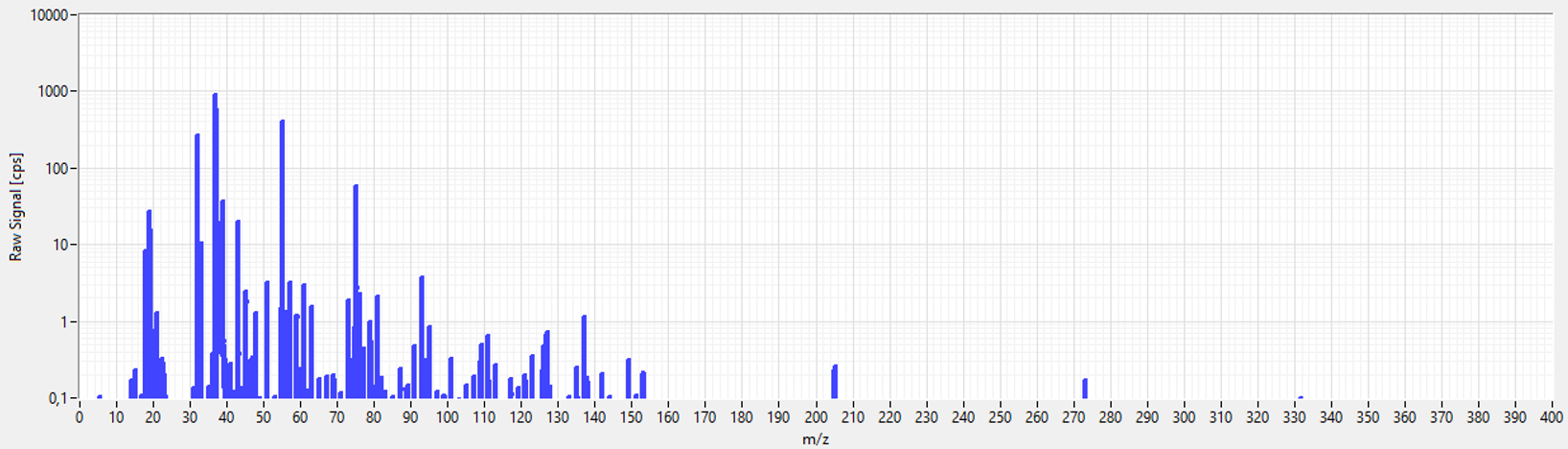

Supplement: S4 Fig — Figure shows an averaged (5 min) spectrum of detected BVOCs of one individual plant in the morning (11 a.m.) before a labelling experiment. Spectra shows uncalibrated, background subtracted values in counts per second (cps) plotted by PTR-MS Viewer 3 (Ionicon Analytic, Innsbruck, Austria). (TIF) [file pone.0204398.s006.tif]
